# Complementary role of transcriptomic endotyping and protein-based biomarkers for risk stratification in sepsis-associated acute kidney injury

**DOI:** 10.1186/s13054-025-05361-3

**Published:** 2025-03-26

**Authors:** Bengi S. Tavris, Christian Morath, Christoph Rupp, Roman Szudarek, Florian Uhle, Timothy E. Sweeney, Oliver Liesenfeld, Mascha O. Fiedler-Kalenka, Simon Dubler, Martin Zeier, Felix C. F. Schmitt, Markus A. Weigand, Thorsten Brenner, Christian Nusshag

**Affiliations:** 1 Department of Nephrology, Heidelberg University Hospital, Heidelberg University, Heidelberg, Germany; 2 Department of Anesthesiology, Heidelberg University Hospital, Heidelberg University, Heidelberg, Germany; 3 SphingoTec GmbH, Hennigsdorf, Berlin, Germany; 4 Clinical Affairs, Inflammatix Inc., Redwood City, USA; 5 Department of Anesthesiology and Intensive Care Medicine, University Hospital Essen, University Duisburg-Essen, Essen, Germany

**Keywords:** Sepsis, Acute kidney injury, Transcriptomic endotyping, Biomarkers, Risk stratification

## Abstract

**Background:**

Sepsis-associated acute kidney injury (SA-AKI) is a prevalent and severe complication in critically ill patients. However, diagnostic and therapeutic advancements have been hindered by the biological heterogeneity underlying the disease. Both transcriptomic endotyping and biomarker profiling have been proposed individually to identify molecular subtypes of sepsis and may enhance risk stratification. This study aimed to evaluate the utility of combining transcriptomic endotyping with protein-based biomarkers for improving risk stratification in SA-AKI.

**Methods:**

This secondary analysis of the PredARRT-Sep-Trial included 167 critically ill patients who met Sepsis-3 criteria. Patients were stratified into three transcriptomic endotypes—inflammopathic (IE), adaptive (AE), and coagulopathic (CE)—using a validated whole-blood gene expression classifier. Eight protein-based biomarkers encompassing kidney function, vascular integrity, and immune response were measured. Predictive performance for the primary endpoint kidney replacement therapy or death was assessed using receiver operating characteristic curve analysis and logistic regression models.

**Results:**

Stratification into transcriptomic endotypes assigned 33% of patients to IE, 42% to AE, and 24% to CE. Patients classified as IE exhibited the highest disease severity and were most likely to meet the primary endpoint (30%), compared to AE and CE (17% and 10%, respectively). Kidney function biomarkers showed stepwise increases with AKI severity across all endotypes, whereas non-functional biomarkers (neutrophil gelatinase-associated lipocalin [NGAL], soluble urokinase plasminogen activator receptor [suPAR], and bioactive adrenomedullin [bio-ADM]) exhibited endotype-specific differences independent of AKI severity. NGAL and suPAR levels were disproportionately elevated in the IE group, suggesting a dominant role of innate immune dysregulation in this endotype. In contrast, bio-ADM, a marker of endothelial dysfunction, was the strongest risk-predictor of outcomes in CE. The combination of transcriptomic endotyping with protein-based biomarkers enhanced predictive accuracy for the primary endpoint and 7-day mortality, with the highest area under the receiver operating characteristic curve of 0.80 (95% CI 0.72–0.88) for endotyping + bio-ADM and 0.85 (95% CI 0.78–0.93) for endotyping and suPAR, respectively. Combinations of endotyping with functional and non-functional biomarkers particularly improved mortality-related risk stratification.

**Conclusions:**

Combining transcriptomic endotyping with protein-based biomarker profiling enhances risk-stratification in SA-AKI, offering a promising strategy for personalized treatment and trial enrichment in the future. Further research should validate these findings and explore therapeutic applications.

**Supplementary Information:**

The online version contains supplementary material available at 10.1186/s13054-025-05361-3.

## Background

Sepsis is characterized by a dysregulated host response to infection, yet the specific mechanisms underlying the immune alterations and subsequent organ dysfunction remain poorly understood [1]. Despite a relatively uniform clinical presentation, the pathophysiology of sepsis is highly heterogeneous, influenced by factors such as genetic variability, comorbidities, and the causative pathogens [1, 2]. This biological diversity complicates both the diagnosis and management of sepsis, contributing to the failure of therapeutic trials [3, 4]. To address this challenge and move beyond the limitations of clinical-based sepsis classifications, current research is focused on stratifying patients according to their underlying pathophysiological drivers using advanced methodologies, including metabolomics, proteomics, and transcriptomics [5]. Recently, several studies have identified distinct sub-phenotypes of acute kidney injury (AKI) in critically ill patients based on circulating levels of new protein-based biomarkers and chemokines, routine laboratory values or patient characteristics. These sub-phenotypes have been linked to varying clinical outcomes and responses to therapy. [6–8]. In contrast to these classification models, which are dependent on clinical variables, transcriptomic endotyping seeks to refine the characterization of sepsis by identifying patients with shared molecular signatures on the basis of gene expression profiles. The identification of these distinct endotypes has significant potential to advance personalized treatment, risk stratification, and our understanding of the complex pathophysiology of sepsis.

Sepsis-associated acute kidney injury (SA-AKI) represents the most common form of AKI in critically ill patients [9–12]. SA-AKI markedly worsens clinical outcomes, prolongs hospital stays, and increases the risk of long-term complications [9]. Despite its substantial global health impact, early diagnostic and therapeutic strategies for SA-AKI remain as limited as those for sepsis overall. Traditional biomarkers such as serum creatinine (SCr) and urinary output (UO) are often delayed in detecting kidney dysfunction and its severity and have limited sensitivity and specificity [13]. Consequently, there is increasing interest in novel biomarkers that can offer earlier, more precise assessment of kidney function impairment and its trajectory.

In this study, we integrated transcriptomic endotyping with a broad panel of protein-based biomarkers relevant to sepsis and kidney dysfunction. Using a previously validated classifier based on the expression of 33 specific mRNAs from whole blood, patients were categorized into three distinct endotypes: inflammopathic (IE), adaptive (AE), and coagulopathic endotype (CE) [5, 14]. These endotypes were initially derived from unsupervised clustering of gene expression data across multiple sepsis cohorts and were named based on subsequent gene ontology analyses reflecting their underlying pathophysiological mechanisms [14].

In addition to SCr, we assessed seven protein-based biomarkers at baseline. Serum cystatin C (CysC) and proenkephalin A (PENK) are alternative markers of kidney function that may offer improved kinetics under non-steady-state conditions, are unaffected by muscle mass, and may provide a more accurate estimation of glomerular filtration rate (GFR) in critically ill patients [15–20]. Neutrophil gelatinase-associated lipocalin (NGAL) has been extensively evaluated as a potential marker of kidney injury [21, 22]. Soluble urokinase plasminogen activator receptor (suPAR) is an immune-derived molecule that is highly expressed in sepsis and has been linked to T-cell-mediated kidney inflammation and adverse kidney-related outcomes [23, 24]. Bioactive adrenomedullin (bio-ADM), an indicator of vascular integrity, is associated with capillary leakage, shock, and the severity of organ dysfunction in sepsis [25, 26]. The combined product of the urinary cell cycle arrest biomarkers tissue inhibitor of metalloproteinase-2 and insulin-like growth factor binding protein 7 ([TIMP2]×[IGFBP7]), serves as an indicator of tubular stress [27]. Finally, kidney injury molecule-1 (KIM-1) in blood is a well-established marker of proximal tubular injury in AKI [27].

Taken together, for the first time, we applied transcriptomic endotyping alongside an endotype-specific assessment of a broad panel of protein-based biomarkers in a cohort of 167 critically ill sepsis patients. We hypothesized that combining transcriptomic endotyping with protein-based biomarker analysis would advance risk-stratification and diagnostic precision to improve outcome prediction for SA-AKI patients by accounting for individual biological drivers of sepsis.

## Methods

### Study design and outcomes

This study is a secondary analysis of the PredARRT-Sep-Trial cohort, a prospective, observational clinical trial conducted at Heidelberg University Hospital, Germany, between May 2017 and September 2019. The trial enrolled adult, critically ill patients who met Sepsis-3 criteria and did not have a prior or immediate need for kidney replacement therapy (KRT), decompensated liver cirrhosis, or a moribund condition at intensive care unit (ICU) admission. At baseline, TEMPUS™ RNA tubes (Applied Biosystems, Foster City, CA, USA) were available for 167 enrolled patients. Protein-based biomarkers were measured in blood or urine at the time of study inclusion (baseline). The primary endpoint of the study was the fulfillment of KRT criteria or death within seven days following sepsis diagnosis. Restrictive criteria for initiating KRT were clearly predefined: serum urea levels exceeding 240 mg/dL, serum potassium levels above 6 mmol/L or persistently above 5.5 mmol/L despite treatment, pH below 7.15 due to pure metabolic or mixed acidosis, acute pulmonary edema caused by fluid overload, requiring either more than 5 L of oxygen to maintain peripheral capillary oxygen saturation (SpO₂) above 95% or a fraction of inspired oxygen (FiO₂) greater than 50%. Patients were classified as KRT, when they developed KRT criteria at any time within 7 days. AKI was defined according to the Kidney Disease: Improving Global Outcomes (KDIGO) criteria, using SCr and UO criteria (worst parameters at any time within seven days after ICU admission), namely mild AKI (KDIGO stage 1), moderate AKI (KDIGO stage 2), and severe AKI (KDIGO stage 3) [28]. More detailed information on the study design, sample collection, laboratory methods and secondary outcomes has been published previously [23].

### RNA isolation, transcriptomic endotyping and biomarker measurement

RNA was extracted using the Tempus™ Spin RNA Isolation Kit (Applied Biosystems, Foster City, CA, USA) following the manufacturer’s protocol. Total RNA concentration was quantified using an RNA-specific fluorometric assay on Qubit platform (Thermo Fisher Scientific, Carlsbad, CA, USA) and its integrity using the 2100 Bioanalyzer System and the corresponding RNA Analysis Kit (Agilent, Santa Clara, CA, USA). Transcriptomic endotyping and assignment to the three predefined endotypes were conducted by Inflammatix (Sunnyvale, CA, USA), blinded to patient data, utilizing whole blood gene expression analysis based on a previously validated classifier [14].

More detailed information on the methodology of transcriptomic endotyping is provided in the additional file.

Protein biomarkers, including suPAR, NGAL, and KIM-1 in blood, as well as [TIMP2]×[IGFBP7] in urine, were measured by experienced technicians using commercially available ELISA kits, as described in our previous study [23]. PENK and bio-ADM levels were quantified by Sphingotec (Hennigsdorf, Berlin, Germany), also blinded to study data. PENK was measured using the sphingotest® penKid® immunoassay (SphingoTec GmbH, Henningsdorf, Berlin, Germany) [29] and bio-ADM using the sphingotest® bio-ADM® immunoassay (SphingoTec GmbH, Henningsdorf, Berlin, Germany). SCr and CysC were analyzed in the accredited central laboratory of Heidelberg University Hospital. Further information on the measurement of biomarkers is provided in the additional file.

### Statistics

SPSS Statistics 28 (IBM) and Prism 10 (GraphPad Software) were used for the statistical analysis. Two-sided P values of less than 0.05 were considered statistically significant. Continuous data were presented as box-and-whisker plots (interquartile range, minimum to maximum) in figures and median with 95%-confidence interval (CI) or interquartile range in tables; categorical data were presented as numbers and percentages. Kruskal-Wallis test was used for multiple-group comparisons, Mann Whitney-U test was used for pairwise comparisons, and Chi-squared test was used for categorical data. Receiver operating characteristics (ROC) analyses and logistic regression models were performed to evaluate the single and combined predictive value of protein-based biomarkers and transcriptomic endotypes. Furthermore, to adjust for potential outcome-relevant confounders, multivariate regression models for the IE were calculated for the primary endpoint, as well as for KRT and death separately. These models included age, gender, chronic kidney disease (CKD), hypertension, and diabetes as covariates.

## Results

### Patient characteristics and outcomes

**Fig. 1:**
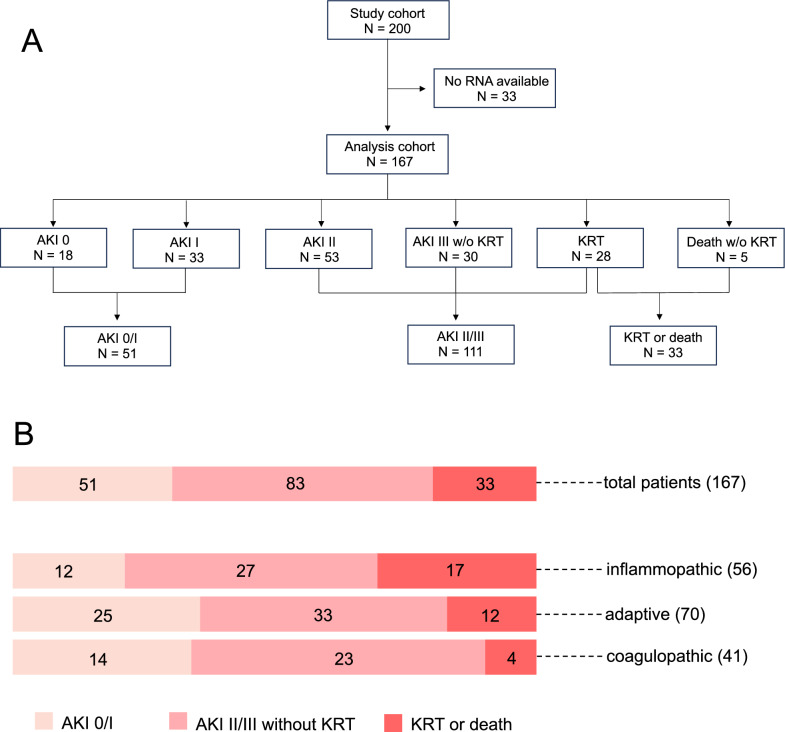
**a** Flow chart of study design and data analysis. Patients were categorized by maximum AKI stage within 7 days. **b** Distribution of AKI stages and transcriptomic endotypes in the analysis cohort

Of the 200 patients initially enrolled in the primary study, 167 were eligible for transcriptomic analyses. Among them, 51 patients (31%) experienced no or mild AKI, 111 patients (66%) presented with moderate or severe AKI, and 33 patients (20%) required KRT or died within seven days of study inclusion (Fig. 1a). Stratification based on transcriptomic endotyping assigned 56 patients (33%) to IE, 70 patients (42%) to AE, and 41 patients (24%) to CE, of which 17 patients (10%), 12 patients (7%) and 4 patients (2%) met the primary endpoint of KRT or death, respectively (Fig. 1b, Table 1).

**Table 1:** Patient characteristics and outcomes stratified by endotypes

Variable	Inflammopathic (n = 56)	Adaptive (n = 70)	Coagulopathic (n = 41)	*p* value
*Demographics*				
Age, years	68.5 (60.3–77)	63 (58.8–72.3)	68 (58.5–74)	0.107
Male sex, n (%)	29 (51.8)	50 (71.4)	27 (65.9)	0.070
BMI, kg/m^2^	27.5 (24.9–30.6)	27.7 (24.3–33.3)	26.9 (23.8–32.3)	0.811
*Preexisting comorbidities*				
Chronic kidney disease (eGFR < 60 ml/min/1,73 m^2), n (%)	10 (17.9)	20 (28.6)	11 (26.8)	0.353
Hypertension, n (%)	44 (78.6)	51 (72.9)	27 (65.6)	0.378
Diabetes mellitus, n (%)	13 (23.2)	25 (35.7)	14 (34.1)	0.287
Coronary heart disease, n (%)	12 (21.4)	14 (20)	4 (9.8)	0.283
Congestive heart failure, n (%)	6 (10.7)	9 (12.9)	5 (12.2)	0.933
Chronic obstructive pulmonary disease, n (%)	6 (10.7)	11 (15.7)	5 (12.2)	0.696
Malignant disease, n (%)	23 (41.1)	31 (44.3)	22 (53.7)	0.453
*Primary source of sepsis*				
Abdomen, n (%)	37 (66.1)	39 (55.7)	31 (75.6)	0.101
Lung, n (%)	13 (23.2)	26 (37.1)	12 (29.3)	0.236
Urinary tract, n (%)	7 (12.5)	7 (10)	1 (2.4)	0.214
Other, n (%)	5 (8.9)	6 (8.6)	1 (2.4)	0.398
*Kidney biomarkers and others*				
Urinary output at inclusion, ml/kg/h	0.7 (0.2–1.5)	0.8 (0.5–1.3)	0.5 (0.3–1.1)	0.174
Baseline serum creatinine, mg/dL	0.8 (0.6–1)	0.9 (0.6–1.2)	0.8 (0.6–1)	0.409
Serum creatinine at inclusion, mg/dL	1.5 (1.1–2.3)	1.5 (0.9–2.6)	1.4 (0.8–1.8)	0.322
Cystatin C at inclusion, mg/L	1.6 (1.2–2.3)	1.8 (1.2–2.6)	1.4 (1–2.1)	0.137
Proenkephalin A at inclusion, pmol/L	76.6 (52.8–102.2)	78.7 (39.3–125.7)	56.9 (35.5–98.2)	0.137
Serum creatinine max, mg /dL	1.8 (1.3–2.8)	1.7 (1.1–3)	1.7 (0.9–2.8)	0.761
Cystatin C max, mg/L	2.1 (1.6–2.8)	2.2 (1.6–3.2)	1.9 (1.2–2.9)	0.324
Proenkephalin A max, pmol/L	85.1 (59.5–142.4)	84.2 (57.9–136.9)	71.3 (40.3–105)	0.124
suPAR at inclusion, ng/mL	8.1 (6.1–13.4)	8.6 (5.3–12.3)	6.7 (4.2–9.7)	**0.010**
NGAL at inclusion, ng/mL	670 (464–1304)	385 (236–619)	503.7 (367.5–741.1)	** < 0.001**
KIM-1 at inclusion, pg/mL	291 (174–414)	228 (166–417)	170 (109–362)	**0.048**
[TIMP2]×[IGFBP7] at inclusion, (ng/mL)^2	1.4 (0.3–3.9)	0.5 (0.2–2.6)	0.7 (0.2–2.3)	0.104
Bio-ADM at inclusion, pg/ml	203 (122–347)	138 (77–291)	136 (78–210)	**0.050**
*Inflammation parameters at inclusion*				
CRP, mg/dL	159 (125–285)	192 (151–277)	221 (160–329)	0.148
PCT, ng/mL	23 (5.7–69.8)	3.3 (1.4–17.6)	5.5 (3–19)	** < 0.001**
Leukocytes, 1/nL	11.2 (5.9–21.1)	15.6 (10.7–25)	11.4 (5.4–20.7)	**0.017**
Thrombocytes, 1/nL	172 (110–322)	230 (112–345)	282 (165–411.5)	0.070
Blood cultures positive, n (%)	24 (42.9)	21 (30)	13 (31.7)	0.086
Gram- bacteria in blood cultures, n (%)	13 (23)	13 (18.6)	9 (22)	0.804
*Sepsis scores*				
SOFA baseline	13 (10.3–15)	11 (8–13)	11 (9–13)	**0.003**
SAPS II baseline	71.5 (64.3–85)	63.5 (45.3–75.3)	68 (50.5–75.5)	**0.014**
SOFA max. (7d)	14 (12–17)	12.5 (9.8–14)	11 (9.5–14)	**0.004**
SAPS II max. (7d)	75 (69.3–87.5)	72 (53.8–84.3)	71 (54.5–82)	0.058
SIC score	3 (2–4)	3 (2–4)	3 (2–4)	0.213
*Outcomes*				
KRT or Death (7d), n (%)	17 (30.4)	12 (17.1)	4 (9.8)	**0.032**
Death (7d), n (%)	10 (17.9)	5 (7.1)	1 (2.4)	**0.026**
Death (30d), n (%)	15 (26.8)	18 (25.7)	5 (12.2)	0.177
Length of ICU stay, days	14 (5–30)	12 (5–32)	9 (4–18)	0.225
Length of hospital stay, days	31 (9.5–66.3)	35 (20.5–63.5)	34 (26.5–52)	0.810
Septic shock, n (%)	54 (96.4)	45 (64.3)	37 (90.2)	** < 0.001**
Hydrocortisone therapy, n (%)	44 (78.6)	44 (62.9)	30 (73.2)	0.144
Invasive ventilation, n (%)	52 (92.9)	57 (81.4)	37 (90.2)	0.129
Cumulative fluid balance in first 24 h, L	3.3 (0.2–6.8)	1.1 (−0.4–3.1)	2.1 (0.3–5.4)	**0.008**

*BMI* body mass index, *suPAR* soluble urokinase plasminogen activator receptor, *NGAL* neutrophil gelatinase-associated lipocalin, *KIM-1* kidney injury molecule 1, *[TIMP-2]*×*[IGFBP7]* the product of tissue inhibitor of metalloproteinases-2 and insulin-like growth factor-binding protein 7 in urine, *bio-ADM* bioactive adrenomedullin, *CRP* C-reactive protein, *PCT* procalcitonin, *SOFA* sepsis-related organ failure assessment score, *SAPS II* simplified acute physiology assessment score II, *SIC* sepsis-induced coagulopathy, *KRT* kidney replacement therapy, *ICU* intensive care unit. Continuous data are presented as median (interquartile range), and categorical data are shown as number (percentage). Bold values indicate statistical significance (*p* ≤ 0.05)

Baseline demographics, pre-existing comorbidities, primary source of sepsis, and kidney function parameters prior to the onset of sepsis were similar across the three endotypes (Table 1). At the time of study inclusion, kidney function markers such as SCr, CysC, and PENK were comparable across all three groups. However, significant differences were observed in non-functional kidney biomarkers and immune- and endothelial-related markers at study inclusion. Specifically, suPAR levels were highest in patients in the IE and AE groups. NGAL levels were especially elevated in the IE and CE groups, KIM-1 levels were elevated in the IE and AE groups, and bio-ADM concentrations were particularly high in the IE group. Conversely, levels of [TIMP2]×[IGFBP7] in urine did not significantly differ across endotypes. Also, routine inflammatory markers and disease severity scores revealed notable differences. Procalcitonin (PCT), sequential organ failure assessment (SOFA) score, and simplified acute physiology score II (SAPS II) were highest in the IE group, while leukocyte counts were most elevated in the AE group. Interestingly, C-reactive protein (CRP) levels, the percentage of patients with positive blood cultures and gram-negative sepsis, the platelet count, and sepsis-induced coagulopathy (SIC) score did not differ significantly between different endotypes at baseline.

The maximum SOFA scores were also highest in patients with IE and AE. In terms of clinical outcomes, patients classified as IE exhibited the highest rates of septic shock, and mortality or need for KRT, compared to those with AE and CE. Even after adjusting for potential outcome-relevant confounders, IE remained the most relevant risk factor for the primary endpoint KRT or death as well as for death and KRT individually with adjusted odds ratios (OR) of 3.40 (95% CI 1.41–8,17), 5.29 (95% CI 1.54–18.20), and 2.80 (95% CI 1.11–7.07), respectively (Table 2, Table S2).

**Table 2:** Multivariable regression analyses adjusted for potential outcome-relevant confounders in the inflammopathic endotype for KRT or death

Variables included	Multivariable OR	95% CI	*p* value
IE	3.40	1.41–8.17	**0.006**
Age	1.01	0.98–1.05	0.516
Male gender	1.45	0.61–3.45	0.401
CKD	3.09	1.28–7.47	**0.012**
Hypertension	0.57	0.22–1.44	0.232
Diabetes	1.17	0.47–2.93	0.735

Bold values indicate statistical significance (*p* ≤ 0.05)

*CI* confidence interval, *CKD* chronic kidney disease, *IE* Inflammopathic endotype, *OR,* odds ratio

### Biomarker levels stratified by endotypes and AKI severity

**Fig. 2:**
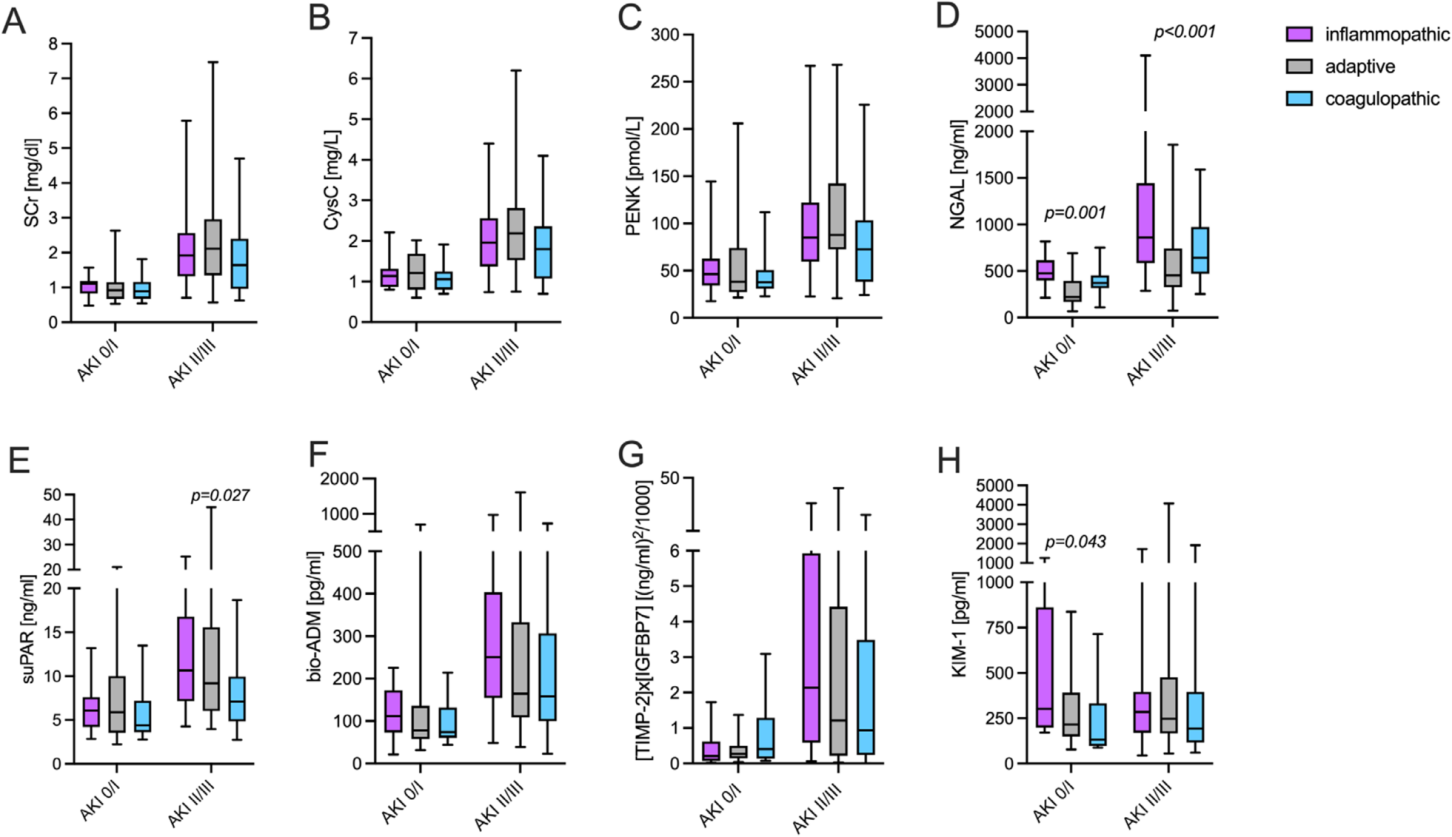
Biomarker levels at baseline stratified by severity of acute kidney injury and endotypes. Data are presented as box-and-whisker plots (interquartile range, minimum to maximum). Upper panel: **A** SCr, serum creatinine; **B** CysC, cystatin C; **C** PENK, proenkephalin A; **D** NGAL, neutrophil gelatinase-associated lipocalin. Lower panel: **E** suPAR, soluble urokinase plasminogen activator receptor; **F** bio-ADM, bioactive adrenomedullin; **G** [TIMP2]×[IGFBP7], the product of urinary tissue inhibitor of metalloproteinases-2 and insulin-like growth factor-binding protein;  **H** KIM-1, kidney injury molecule-1. AKI, acute kidney injury

Next, we analyzed kidney biomarker levels stratified by both AKI severity and transcriptomic endotypes (Fig. 2). Except for KIM-1, all biomarkers showed a stepwise increase with rising AKI severity, independent of the respective endotype.

We further identified endotype-specific variations in biomarker levels within the same AKI severity group. NGAL levels were notably higher in patients with IE in relation to those with AE and CE, particularly in individuals with moderate or severe AKI (Fig. 2d). Additionally, suPAR levels were shown to be higher in patients with IE and AE endotypes compared to patients with CE (Fig. 2e).

### Stratification by biomarker quartiles and endotypes

**Fig. 3:**
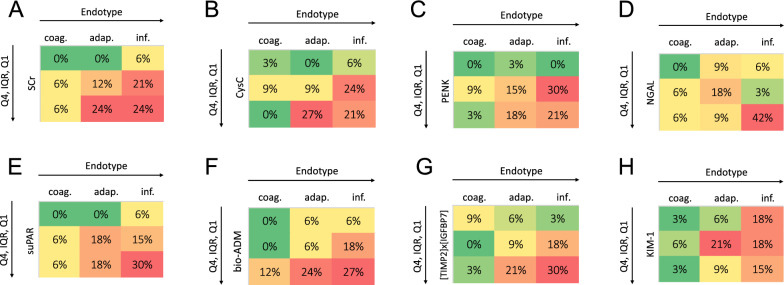
Patients who died or required kidney replacement therapy (KRT) within seven days stratified by endotypes and biomarker quartiles. Data are shown as heatmaps. The percentage of patients in the total cohort who received KRT or died (n = 33) is shown in each cell. Left to right: coagulopathic, adaptive, inflammopathic endotype; top to bottom: first quartile, interquartile range, fourth quartile of biomarker values in the entire cohort. Upper panel: **A** SCr, serum creatinine; **B** CysC, cystatin C; **C** PENK, proenkephalin A; **D** NGAL, neutrophil gelatinase-associated lipocalin. Lower panel: **E** suPAR, soluble plasminogen activator receptor; **F** bio-ADM, bioactive adrenomedullin; **G** [TIMP2]×[IGFBP7], the product of urinary tissue inhibitor of metalloproteinases-2 and insulin-like growth factor-binding protein; **H** KIM-1, kidney injury molecule-1. IQR, interquartile range

To gain a clearer understanding of the differences in the risk stratification of analyzed biomarkers for distinct endotypes, we stratified patients who either met the primary endpoint of KRT or death (Fig. 3), or both outcomes individually (Figure S1, S2), into transcriptomic endotypes and biomarker quartiles.

For the functional biomarkers SCr, CysC, and PENK, patients classified as AE or IE demonstrated the expected association between higher biomarker levels and an increased likelihood of reaching the primary endpoint of KRT or death (Fig. 3a–c). A similar trend was observed for the non-functional biomarkers suPAR, bio-ADM, and [TIMP2]×[IGFBP7] in the AE and IE groups (Fig. 3e–g). Similar trends were observed for the outcome death or KRT alone (Figure S1, S2).

NGAL performed particularly well in patients classified as IE, where those in the upper quartile of NGAL levels showed the highest incidence of KRT or death. This observation was even clearer, when the same analysis was performed solely for the endpoint KRT (Figure S2). In contrast, elevated NGAL levels in the AE or CE group did not effectively risk stratify patients for the primary endpoint, or KRT or death individually (Fig. 3d, Figure S1, S2). Surprisingly, in patients classified as CE, elevated levels of CysC and PENK did not correlate with the endpoints tested, while SCr, suPAR, and NGAL showed a moderate predictive value for risk stratification (Fig. 3d, Figure S1, S2). Bio-ADM levels, however, provided the best risk stratification for within the CE group, where most other biomarkers failed (Fig. 3f, Figure S1, S2). KIM-1, on the other hand, exhibited a poor association between elevated biomarker levels and adverse outcomes across all three endotypes and outcomes (Fig. 3h, Figure S1, S2).

### Predictive performance using endotypes combined with protein-based biomarkers

To further evaluate the diagnostic validity, we conducted ROC analyses of various biomarkers and their combinations at baseline to predict the primary endpoint of KRT or death in seven days (Table 3), or death (Table S3) and KRT individually (Table S4). Among the individual biomarkers, suPAR demonstrated the highest area under the curve (AUC), followed by the functional biomarkers CysC and PENK each with AUC values exceeding 0.75 for the primary endpoint. In contrast, KIM-1, [TIMP2]x[IGFBP7] and SCr exhibited the lowest predictive performance. Predictive accuracy was enhanced for certain combinations when biomarkers were combined with transcriptomic endotyping. The highest AUCs were observed when endotyping was combined with bio-ADM, suPAR or CysC (Table 3). Furthermore, combining endotyping with both functional and non-functional biomarkers yielded AUCs mostly exceeding 0.80 for predicting KRT or death within seven days (Table 3). However, when solely functional biomarkers were combined with the top three non-functional biomarkers (bio-ADM, suPAR, and NGAL), the predictive performance for the primary endpoint was largely comparable to that achieved by combining functional and non-functional biomarkers with endotyping (Table 3).

**Table 3:** Receiver operating characteristic analyses for KRT or death within seven days

Biomarker alone	AUC (95% CI)	Biomarker + endotyping	AUC (95% CI)
SCr	0.73 (0.64–0.83)	SCr + endotyping	0.76 (0.66–0.86)
CysC	0.75 (0.66–0.84)	CysC + endotyping	0.78 (0.69–0.87)
PENK	0.75 (0.66–0.84)	PENK + endotyping	0.77 (0.69–0.86)
NGAL	0.74 (0.63–0.85)	NGAL + Endotyping	0.75 (0.64–0.86)
suPAR	0.76 (0.67–0.85)	suPAR + endotyping	0.78 (0.69–0.87)
bio-ADM	0.74 (0.64–0.85)	bio-ADM + endotyping	0.80 (0.72–0.88)
[TIMP2]×[IGFBP7]	0.71 (0.60–0.83)	[TIMP-2]×[IGFBP7] + endotyping	0.73 (0.63–0.84)
KIM-1	0.50 (0.38–0.61)	KIM-1 + endotyping	0.66 (0.56–0.77)

Functional + Non-functional biomarker	AUC (95% CI)	Functional + non-functional biomarker + endotyping	AUC (95% CI)
SCr + bio-ADM	0.82 (0.73–0.90)	SCr + bio-ADM + endotyping	0.84 (0.78–0.91)
SCr + suPAR	0.81 (0.73–0.90)	SCr + suPAR + endotyping	0.82 (0.75–0.90)
SCr + NGAL	0.81 (0.72–0.9)	SCr + NGAL + endotyping	0.81 (0.73–0.9)
CysC + bio-ADM	0.80 (0.72–0.89)	CysC + bio-ADM + endotyping	0.84 (0.76–0.90)
CysC + suPAR	0.78 (0.70–0.87)	CysC + suPAR + endotyping	0.81 (0.73–0.89)
CysC + NGAL	0.79 (0.7–0.89)	CysC + NGAL + endotyping	0.79 (0.7–0.88)
PENK + bio-ADM	0.82 (0.75–0.90)	PENK + bio-ADM + endotyping	0.84 (0.78–0.91)
PENK + suPAR	0.82 (0.74–0.89)	PENK + suPAR + endotyping	0.83 (0.76–0.90)
PENK + NGAL	0.80 (0.72–0.89)	PENK + NGAL + endotyping	0.80 (0.71–0.89)

*SCr* serum creatinine, *CysC* cystatin C, *PENK* proenkephalin A, *NGAL* neutrophil gelatinase-associated lipocalin, *suPAR* soluble urokinase plasminogen activator receptor, *bio-ADM* bioactive adrenomedullin, *[TIMP-2]*×*[IGFBP7]* the product of tissue inhibitor of metalloproteinases-2 and insulin-like growth factor-binding protein 7 in urine, *KIM-1* kidney injury molecule 1

For the specific outcome death within seven days, combining endotyping with individual biomarkers resulted in a greater improvement in nominal AUC values compared to the primary outcome of KRT or death (Table S3). Furthermore, unlike the ROC analyses for the primary endpoint (Table 3), the combination of endotyping with both functional and non-functional biomarkers enhanced nominal AUCs for predicting death, compared to using functional and non-functional biomarkers alone (Table S3). The highest AUC, 0.88, was achieved by combining PENK and suPAR with endotyping. ROC characteristics for death and KRT within seven days as separate outcomes are presented in Table S3 and S4 in the additional file.

## Discussion

SA-AKI and its associated complications remain a significant challenge in critical care medicine due to their complex and multifaceted nature. Despite advances in our understanding of the pathophysiology of sepsis, diagnostic and therapeutic strategies have lagged, largely due to the biological heterogeneity of the condition. This diversity highlights the need for innovative approaches that move beyond traditional "one-size-fits-all" frameworks, currently applied to sepsis patients and to established and emerging protein-based biomarkers. In this study, we sought to address these challenges by integrating transcriptomic endotyping with biomarker profiling to provide a novel perspective on the molecular driver-dependent role of biomarkers for risk stratification of SA-AKI. Our findings may provide a deeper understanding of the interplay between the type of immune dysregulation, endothelial dysfunction, and kidney injury and shed light on the potential for more precise and personalized management strategies using biomarkers together with transcriptomic endotyping.

In this context, we now observed distinct pathophysiological processes of the respective endotype to be associated with certain biomarker profiles across the three tested endotypes. IE was associated with the worst outcomes, a finding consistent with earlier studies linking systemic inflammation to poor sepsis outcomes [14, 30]. Patients assigned to IE had the highest SOFA scores, the highest incidence of KRT or death, and septic shock, as well as pronounced proinflammatory biomarker levels, such as NGAL and PCT. This may specifically underscore the role of excessive activation of the innate immune system in disease progression in this group. Thus, NGAL, secreted by neutrophils after activation, most likely reflects both tubular injury and systemic inflammatory activity in our study [21, 31, 32]. Especially its consistently elevated levels in the IE group may suggest the predominant role of innate immune system dysregulation in driving poor outcomes and explains its relevance for outcome prediction, especially for IE. Similarly, suPAR, a marker of immune activation, was significantly increased in IE, suggesting neutrophil-mediated inflammation once more as a critical mechanism, since suPAR is another protein released by neutrophils under inflammatory conditions [33]. Importantly, these findings align with the gene ontology analyses of Sweeney et al., which associate IE with pro-inflammatory signaling pathways of innate immune response [14]. Furthermore, except for KIM-1, all biomarker levels increased stepwise with rising AKI severity, but NGAL and suPAR levels were disproportionately higher, particularly in IE patients across all AKI stages. This suggests again that systemic inflammation, rather than kidney injury alone, drives the elevation of these biomarkers in this endotype. The high value of NGAL and suPAR for risk-stratification for the primary endpoint KRT or death within the IE group—but also for the outcome death or KRT individually—supports their role as valuable, context-dependent biomarkers for this specific endotype. On the other hand, the specific pathophysiological triggers of KIM-1 expression, which is primarily a marker of proximal tubular injury, may not fully align with the systemic inflammatory and vascular dysfunction mechanisms observed in early sepsis, potentially explaining its poor diagnostic performance [34].

AE is characterized by the dominance of transcripts mainly related to adaptive immune response [14]. Adaptive immunity is known to be activated during prolonged sepsis, driven by damage-associated molecular patterns (DAMPs) and pathogen-associated molecular patterns (PAMPs) released during innate immune activation [35–37]. This immune activation may contribute to the relatively low early mortality in the AE group (7.1%) but higher 30-day mortality (25.7%), possibly due to T-cell depletion and susceptibility to secondary infections [2, 33, 38]. In contrast to NGAL, suPAR also showed value for risk stratification in AE. This may be attributed to suPAR’s role in linking innate immune activation to adaptive immune response, as shown recently by our group, connecting high blood suPAR levels to T-cell-mediated kidney tissue inflammation and damage in experimental sepsis [23].

In contrast to IE, CE was associated with the lowest rates of KRT and mortality, in line with findings from Balch et al. but inconsistent with earlier reports suggesting worse outcomes in CE [14, 39]. This difference may be explained by multiple factors. First, Sweeney et al. report that the greatest inaccuracy of the classifier lies in the differentiation of IE from CE, which may affect the incidence of certain outcomes associated with a specific endotype [14]. Second, there were relevant differences in patient characteristics, individual disease severity and incidence of disseminated intravascular coagulation (DIC)/extent of coagulopathy between study cohorts. For instance, in the original study by Sweeney et al., patients with CE were significantly older compared to IE and AE, with a median age of 49.7, 34.8 and 38.5, respectively [14]. On the other hand, although our cohort was older, with an overall median age of 67, there were no significant age differences across endotypes. Hence, age differences may act as a confounding factor in this context. Third, in the study by Sweeney et al., patients with CE showed an overall higher disease severity compared to other endotypes. In contrast, we observed a higher disease severity in patients assigned to IE and AE compared to CE (maximum SOFA scores, Table 1). Lastly, in the Sweeney study, patients assigned to CE had significantly higher rates of DIC, which in general is associated with poorer outcomes [40]. In contrast, the severity of coagulopathy was not different across endotypes in our study, when looking at SIC scores [41].

The strong performance of bio-ADM in risk-stratifying outcomes in CE potentially highlights the role of endothelial dysfunction and vascular leakage in this endotype. However, bio-ADM, a key regulator of vascular integrity, was consistently associated with meeting the primary endpoint, or KRT or death individually, across all endotypes, suggesting that endothelial barrier disruption may be a universal contributor to disease severity and SA-AKI progression [25, 42].

Notably, while high SCr levels are traditionally associated with poor outcomes in sepsis, this relationship held especially true for IE and AE, but less for CE. These finding challenges the conventional understanding and highlights the need to consider underlying pathophysiological drivers when interpreting biomarker data. In summary, our findings suggest that the pathophysiological basis of SA-AKI varies significantly among endotypes, influencing the performance of specific protein-based biomarkers.

Lastly, the combination of protein-based biomarkers with transcriptomic endotyping enhanced predictive accuracy for specific combinations and tested outcomes. Combining functional and non-functional biomarkers with endotyping yielded AUC values exceeding 0.80. This approach aligns with the concepts from the 23rd Acute Disease Quality Initiative Consensus Conference, which advocates combining functional and damage-related kidney markers to improve diagnostic accuracy in AKI [13]. However, when analyzing the outcomes KRT or death separately across all endotypes, it became clear that the nominal improvements in AUC values were primarily relevant for the outcome death. Nonetheless, this does not diminish the significance of our findings, as understanding how different endotypes and biomarkers interact—and identifying potential redundancies—is a crucial step toward advancing personalized sepsis care.

Furthermore, our analyses show that the use of ROC analyses alone to assess the validity and clinical relevance of biomarkers provides an incomplete picture. By examining biomarker quartiles within specific endotypes, we identified distinct biomarker patterns that offer valuable insights for risk stratification within a given endotype and for all outcomes tested in our study, that may alter the interpretation of biomarker data in the future.

In summary, transcriptomic endotyping seems to further improve risk stratification in patients with SA-AKI by accounting for the biological heterogeneity of sepsis and may offer a promising tool for trial enrichment and more personalized therapeutic interventions in the future.

An example in this context is the timing of KRT. Multiple randomized controlled trials have shown that early initiation of KRT in both septic and non-septic patients does not improve outcomes [43, 44]. However, when late KRT initiation strategies are applied, approximately 50% of patients recover sufficient kidney function and do not require KRT at all—these patients exhibit the lowest mortality rates [43–45]. In contrast, patients whose kidney function continues to deteriorate develop severe metabolic disturbances and emergency indications for KRT, which are associated with the highest mortality rates [43–47]. This suggests that while some patients benefit from delayed KRT to allow for kidney recovery, others may require earlier initiation to prevent reaching critical thresholds.

In this context, patient-centered endotyping combined with protein-based biomarkers at ICU admission could help identify individuals at high risk of progressing to late-stage (emergency) KRT criteria, as demonstrated in our study. This approach could optimize KRT timing for this subgroup, potentially preventing critical fluid overload and severe metabolic disturbances, both of which are consistently linked to poor outcomes in critically ill patients [48–50].

Another promising application of endotyping combined with protein-based markers is the potential for individualized hydrocortisone therapy in patients with septic shock. Yao et al. demonstrated that hydrocortisone therapy increases 28-day mortality in septic patients with high adaptive immune activity, whereas it may potentially have a beneficial effect in those with predominant innate immune activity [51]. Thus, identifying patients with dominant innate immune activation through transcriptomic endotyping could help tailor hydrocortisone and vasopressor therapy, to improve outcomes such as AKI severity and overall mortality. However, this concept remains hypothetical at present, and future clinical trials are necessary to explore its clinical relevance and effectiveness.

This study has several limitations. First, it was a secondary analysis of a single-center study with a limited sample size, which may affect the generalizability of the findings, especially in smaller subgroup analysis. Nevertheless, our data provide valuable first insights in the complementary role of transcriptomic endotyping and protein-based biomarkers that could help shaping future research directions. Moreover, the distribution of patients across endotypes in our cohort closely mirrors that reported in the original work by Sweeney et al., suggesting good reproducibility even in this small ICU cohort [14]. Second, endotyping was performed only at baseline and was not repeated during the study period. Changes in endotype classification during disease progression, as reported by Kyriazopoulou et al., could influence the observed associations [30]. Nonetheless, since biomarker levels were measured concurrently with endotyping, the biomarker variations likely reflect real differences between the endotypes at this point in time. Finally, while we discuss potential underlying pathophysiological mechanisms based on observed biomarker patterns and known biology, the findings represent associations rather than causal relationships in our study. Future mechanistic studies are required to validate these observations and elucidate the specific pathways involved in SA-AKI.

## Conclusions

In this secondary analysis of a prospective observational study, transcriptomic endotyping emerged as a promising tool for risk stratification and patient enrichment for future studies investigating novel therapeutic approaches in SA-AKI. IE was associated with the most severe disease course and the highest rates of KRT and death. Further, the integration of transcriptomic endotyping with solely protein-based biomarkers enhanced individual risk stratification for certain combinations and outcomes. However, incorporating gene expression-based endotyping into combinations of functional and non-functional biomarkers added little to prognostic accuracy (in terms of AUC) with regard to the primary endpoint KRT or death, or the endpoint KRT alone. Nevertheless, this approach may hold potential for predicting mortality, a hypothesis that requires confirmation in larger studies.

By stratifying patients into distinct molecular endotypes and evaluating a comprehensive panel of kidney, vascular and immune-related biomarkers, we identified meaningful associations between specific endotypes, different biomarker biologies, and clinical outcomes. This integrative approach highlights the heterogeneity of SA-AKI and emphasizes the importance of tailoring diagnostic and therapeutic strategies to individual pathophysiological drivers.

## Supplementary Information

Additional file 1

## Data Availability

The datasets used and/or analyzed during the current study are available from the corresponding author upon reasonable request.

## Abbreviations

AE: Adaptive endotype
AUC: Area under the curve
Bio-ADM: Bioactive adrenomedullin
CE: Coagulopathic endotype
CI: Confidence interval
CKD: Chronic kidney disease
CRP: C-reactive protein
CysC: Cystatin C
DAMP: Damage-associated molecular pattern
DIC: Disseminated intravasal coagulopathy
GFR: Glomerular filtration rate
ICU: Intensive care unit
IE: Inflammopathic endotype
KDIGO: Kidney disease: improving global outcomes
KIM-1: Kidney injury molecule-1
KRT: Kidney replacement therapy
NGAL: Neutrophil gelatinase-associated lipocalin
OR: Odds ratio
PAMP: Pathogen-associated molecular pattern
PCT: Procalcitonin
PENK: Proenkephalin A
ROC: Receiver operating characteristics
SA-AKI: Sepsis associated acute kidney injury
SAPS II: Simplified Acute Physiology Score II
SCr: Serum creatinine
SIC: Sepsis-induced coagulopathy
SOFA: Sequential organ failure assessment
SuPAR: Soluble urokinase plasminogen activator receptor
[TIMP2]×[IGFBP7]: The product of urinary tissue inhibitor metalloproteinase-2 and insulin-like growth factor-binding protein 7
UO: Urine output
