# Supplementary material for: Complementary role of transcriptomic endotyping and protein-based biomarkers for risk stratification in sepsis-associated acute kidney injury
Source: Crit Care. 2025 Mar 26;29:136. doi: 10.1186/s13054-025-05361-3 (PMC11948859; doi:10.1186/s13054-025-05361-3)
Supplement: Supplementary file 1 — Additional file 1 [file 13054_2025_5361_MOESM1_ESM.pdf]

Supplementary material

Complementary role of transcriptomic endotyping and protein-based biomarkers for risk stratification in sepsis-associated acute kidney injury

Bengi S. Tavris, Department of Nephrology, Heidelberg University Hospital, Heidelberg University, Heidelberg, Germany

Christian Morath, Department of Nephrology, Heidelberg University Hospital, Heidelberg University, Heidelberg, Germany

Christoph Rupp, Department of Anesthesiology, Heidelberg University Hospital, Heidelberg University, Heidelberg, Germany

Roman Szudarek, Department of Anesthesiology, Heidelberg University Hospital, Heidelberg University, Heidelberg, Germany

Florian Uhle, Department of Anesthesiology, Heidelberg University Hospital, Heidelberg University, Heidelberg, Germany and SphingoTec GmbH, Hennigsdorf, Berlin, Germany

Timothy E. Sweeney, Clinical Affairs, Inflammatix Inc., RedwoodCity, USA

Oliver Liesenfeld, Clinical Affairs, Inflammatix Inc., RedwoodCity, USA

Mascha O. Fiedler-Kalenka, Department of Anesthesiology, Heidelberg University Hospital, Heidelberg University, Heidelberg, Germany

Simon Dubler, Department of Anesthesiology and Intensive Care Medicine, University Hospital Essen, University Duisburg-Essen, Essen, German

Martin Zeier, Department of Nephrology, Heidelberg University Hospital, Heidelberg University, Heidelberg, Germany

Felix C.F. Schmitt, Department of Anesthesiology, Heidelberg University Hospital, Heidelberg University, Heidelberg, Germany

Markus A. Weigand, Department of Anesthesiology, Heidelberg University Hospital, Heidelberg University, Heidelberg, Germany

Thorsten Brenner, Department of Anesthesiology and Intensive Care Medicine, University Hospital Essen, University Duisburg-Essen, Essen, German

Christian Nusshag, Department of Nephrology, Heidelberg University Hospital, Heidelberg University, Heidelberg, Germany

Correspondence to:

Christian Nusshag

Department of Nephrology, Germany

University Hospital Heidelberg

Im Neuenheimer Feld 162, 69120 Heidelberg

Phone: (+49) 6221-9112-0

Email: Christian.Nusshag@med.uni-heidelberg.de

Table of contents:

Supplementary methods

Methodology of Transcriptomic endotyping

Biomarker measurements

Supplementary tables and figures

Supplementary methods

Methodology of transcriptomic endotyping

The genes used for endotyping are listed below and categorized into six different modules under three different clusters representing the three endotypes: IE, AE and CE (Table S1). Here, three endotypes each have an 'up' and 'down' module. The geometric mean of 'down' genes is subtracted by the geometric mean of 'up' genes to calculate a score for each endotype, generating three probabilities for each sample. In the next step, a three-class logistic regression model accounts for these three probabilities and produces a final endotype prediction. The endotyping methodology is described in more detail in the original paper by Sweeney et al. (1).

Biomarker measurements

All samples were thawed only once for the respective biomarker measurements. KIM-1, NGAL, suPAR, penKID and bio-ADM were measured in duplicates using commercially available ELISA kits. The sphingotest® bio-ADM® indicates repeatability of below ±10% coefficient of variation (CV) for bio-ADM concentrations from 12 pg/mL to 352 pg/mL. The sphingotest® penKid® indicates repeatability of below ±10 % CV for penKid concentrations from 50 pmol/L to 1 000 pmol/L. The bioporto diagnostics human NGAL ELISA Kit indicates repeatability of 4-14% CV for NGAL concentrations from 25 ng/ml to 3490 ng/ml in plasma. Quantikine Elisa Human Serum KIM-1 Immunoassay indicates repeatability of below 7% CV for KIM-1. SuPAR was measured using the Virogates suPARnostic Elisa Kit. The kit indicates

repeatability of below 10% CV for suPAR levels from 0.4 ng/ml to 16 ng/ml. All samples with a CV >20% were remeasured according to internal standards.

The product of TIMP2 and IGFBP7 ([TIMP2]x[IGFBP7]) was measured using an FDA-approved point-of-care device called the NEPHROCHECK® Test System. The NEPHROCHECK® Test reports the product of both biomarkers with an imprecision of ≤15% within-run CV.

Supplementary tables and figures

Table S1: Genes related to the respective transcriptomic endotypes

Inflammopathic (IE)		Adaptive (IE)		Coagulopathic (CE)	
Up	Down	Up	Down	Up	Down
ARG1<br>LCN2<br>LTF<br>OLFM4	HLA-DMB	YKT6<br>PDE4B<br>TWISTNB<br>BTN2A2<br>ZBTB33<br>PSMB9<br>CAMK4<br>TMEM19<br>SLC12A7<br>TP53BP1<br>PLEKHO1<br>SLC25A22<br>FRS2	GADD45A<br>CD24<br>S100A12<br>STX1A	KCNMB4<br>CRISP2<br>HTRA1<br>PPL	RHBDF2<br>ZCCHC4<br>YKT6<br>DDX6<br>SENP5<br>RAPGEF1<br>DTX2<br>RELB

ARG1 (Arginase 1), LCN2 (Lipocalin 2), LTF (Lactotransferrin), OLFM4 (Olfactomedin 4), HLA-DMB (Major Histocompatibility Complex, Class II, DM Beta), YKT6 (YKT6 v-SNARE Homolog), PDE4B (Phosphodiesterase 4B), TWISTNB (TWIST Neighbor), BTN2A2 (Butyrophilin Subfamily 2 Member A2), ZBTB33 (Zinc Finger and BTB Domain Containing 33), PSMB9 (Proteasome Subunit Beta 9), CAMK4 (Calcium/Calmodulin-Dependent Protein Kinase IV), TMEM19 (Transmembrane Protein 19), SLC12A7 (Solute Carrier Family 12 Member 7), TP53BP1 (Tumor Protein P53 Binding Protein 1), PLEKHO1 (Pleckstrin Homology Domain Containing O1), SLC25A22 (Solute Carrier Family 25 Member 22), FRS2 (Fibroblast Growth Factor Receptor Substrate 2), GADD45A (Growth Arrest and DNA Damage Inducible Alpha), CD24 (CD24 Molecule), S100A12 (S100 Calcium Binding Protein A12), STX1A (Syntaxin 1A), KCNMB4 (Potassium Calcium-Activated Channel Subfamily M Regulatory Beta Subunit 4), CRISP2 (Cysteine-Rich Secretory Protein 2), HTRA1 (HtrA Serine Peptidase 1), PPL (Periplakin), RHBDL2 (Rhomboid Like 2), ZCCHC4 (Zinc Finger CCHC-Type Containing 4), YKT6 (YKT6 v-SNARE Homolog), DDX6 (DEAD-Box Helicase 6), SENP5 (SUMO Specific Peptidase 5), RAPGEF1 (Rap Guanine Nucleotide Exchange Factor 1), DTX2 (Deltex E3 Ubiquitin Ligase 2), RELB (RELB Proto-Oncogene, NF-KB Subunit).

Table S2: Multivariable regression analyses adjusted for potential outcome-relevant confounders in the inflammopathic endotype for KRT or death alone.

Variables included	Multivariable OR	95 % CI	p-value
**Model for death within seven days**			
**IE**	5.29	1.54-18.20	0.008
**Age**	1.03	0.98-1.09	0.298
**Male gender**	0.92	0.29-2.90	0.882
**CKD**	3.67	1.11-12.16	0.033
**Hypertension**	0.36	0.10-1.33	0.123
**Diabetes**	2.84	0.84-9.63	0.095
**Model for KRT within seven days**			
**IE**	2.80	1.11-7.07	0.030
**Age**	1.00	0.96-1.04	0.942
**Male gender**	1.78	0.69-4.51	0.238
**CKD**	3.28	1.33-8.12	0.010
**Hypertension**	0.78	0.28-2.18	0.635
**Diabetes**	1.17	0.45-3.05	0.745

CI, confidence interval, CKD, chronic kidney disease; IE, inflammopathic endotype, OR, odds ratio.

Table S3: Receiver operating characteristic analyses for death within seven days.

Biomarker alone	AUC (95% CI)	Biomarker + Endotyping	AUC (95% CI)
SCr	0.66 (0.54-0.78)	SCr + Endotyping	0.74 (0.62-0.86)
CysC	0.70 (0.58-0.82)	CysC + Endotyping	0.76 (0.65-0.87)
PENK	0.67 (0.54-0.80)	PENK + Endotyping	0.73 (0.60-0.86)
NGAL	0.69 (0.51-0.87)	NGAL + Endotyping	0.75 (0.61-0.89)
suPAR	0.81 (0.71-0.92)	suPAR + Endotyping	0.85 (0.78-0.93)
bio-ADM	0.69 (0.53-0.84)	bio-ADM + Endotyping	0.78 (0.69-0.87)
[TIMP2]x[IGFBP7]	0.64 (0.48-0.80)	[TIMP-2]x[IGFBP7] + Endotyping	0.73 (0.58-0.87)
KIM-1	0.47 (0.32-0.63)	KIM-1 + Endotyping	0.71 (0.58-0.85)
**Functional + Non-functional Biomarker**	**AUC (95% CI)**	**Functional + Non-functional Biomarker + Endotyping**	**AUC (95% CI)**
SCr + bio-ADM	0.71 (0.56-0.85)	SCr + bio-ADM + Endotyping	0.78 (0.69-0.88)
SCr + suPAR	0.81 (0.70-0.91)	SCr + suPAR + Endotyping	0.85 (0.78-0.93)
SCr + NGAL	0.72 (0.55-0.88)	SCr + NGAL + Endotyping	0.76 (0.64-0.89)
CysC + bio-ADM	0.72 (0.58-0.86)	CysC + bio-ADM + Endotyping	0.80 (0.71-0.89)
CysC + suPAR	0.81 (0.71-0.92)	CysC + suPAR + Endotyping	0.86 (0.79-0.93)
CysC + NGAL	0.74 (0.58-0.89)	CysC + NGAL + Endotyping	0.79 (0.67-0.90)
PENK + bio-ADM	0.76 (0.65-0.87)	PENK + bio-ADM + Endotyping	0.80 (0.71-0.89)
PENK + suPAR	0.84 (0.76-0.93)	PENK + suPAR + Endotyping	0.88 (0.82-0.94)
PENK + NGAL	0.74 (0.58-0.89)	PENK + NGAL + Endotyping	0.77 (0.64-0.90)

SCr, serum creatinine; CysC, cystatin C; PENK, proenkephalin A; NGAL, neutrophil gelatinase-associated lipocalin; suPAR, soluble urokinase plasminogen activator receptor; bio-ADM, bioactive adrenomedullin; [TIMP-2]x[IGFBP7], the product of tissue inhibitor of metalloproteinases 2 and insulin-like growth factor-binding protein 7 in urine; KIM-1, kidney injury molecule 1.

Table S4: Receiver operating characteristic analyses for KRT within seven days.

**Biomarker alone**	**AUC (95% CI)**	**Biomarker + Endotyping**	**AUC (95% CI)**
SCr	0.78 (0.69-0.87)	SCr + Endotyping	0.78 (0.69-0.88)
CysC	0.77 (0.69-0.86)	CysC + Endotyping	0.77 (0.67-0.87)
PENK	0.73 (0.64-0.83)	PENK + Endotyping	0.74 (0.64-0.83)
NGAL	0.77 (0.66-0.89)	NGAL + Endotyping	0.76 (0.64-0.88)
suPAR	0.78 (0.69-0.88)	suPAR + Endotyping	0.78 (0.69-0.88)
bio-ADM	0.80 (0.70-0.91)	bio-ADM + Endotyping	0.84 (0.75-0.92)
[TIMP2]x[IGFBP7]	0.73 (0.64-0.83)	[TIMP-2]x[IGFBP7] + Endotyping	0.73 (0.61-0.84)
KIM-1	0.51 (0.39-0.63)	KIM-1 + Endotyping	0.62 (0.50-0.74)
**Functional + Non-functional Biomarker**	**AUC (95% CI)**	**Functional + Non-functional Biomarker + Endotyping**	**AUC (95% CI)**
SCr + bio-ADM	0.88 (0.82-0.94)	SCr + bio-ADM + Endotyping	0.88 (0.72-0.94)
SCr + suPAR	0.85 (0.76-0.93)	SCr + suPAR + Endotyping	0.85 (0.77-0.93)
SCr + NGAL	0.85 (0.77-0.93)	SCr + NGAL + Endotyping	0.85 (0.77-0.93)
CysC + bio-ADM	0.86 (0.79-0.93)	CysC + bio-ADM + Endotyping	0.87 (0.80-0.93)
CysC + suPAR	0.80 (0.71-0.89)	CysC + suPAR + Endotyping	0.81 (0.72-0.90)
CysC + NGAL	0.82 (0.73-0.91)	CysC + NGAL + Endotyping	0.82 (0.73-0.91)
PENK + bio-ADM	0.85 (0.77-0.92)	PENK + bio-ADM + Endotyping	0.86 (0.79-0.93)
PENK + suPAR	0.82 (0.73-0.90)	PENK + suPAR + Endotyping	0.82 (0.75-0.90)
PENK + NGAL	0.80 (0.70-0.90)	PENK + NGAL + Endotyping	0.80 (0.70-0.90)

SCr, serum creatinine; CysC, cystatin C; PENK, proenkephalin A; NGAL, neutrophil gelatinase-associated lipocalin; suPAR, soluble urokinase plasminogen activator receptor; bio-ADM, bioactive adrenomedullin; [TIMP-2]x[IGFBP7], the product of tissue inhibitor of metalloproteinases 2 and insulin-like growth factor-binding protein 7 in urine; KIM-1, kidney injury molecule 1.

Stratification for death within seven days by biomarker quartiles and endotypes

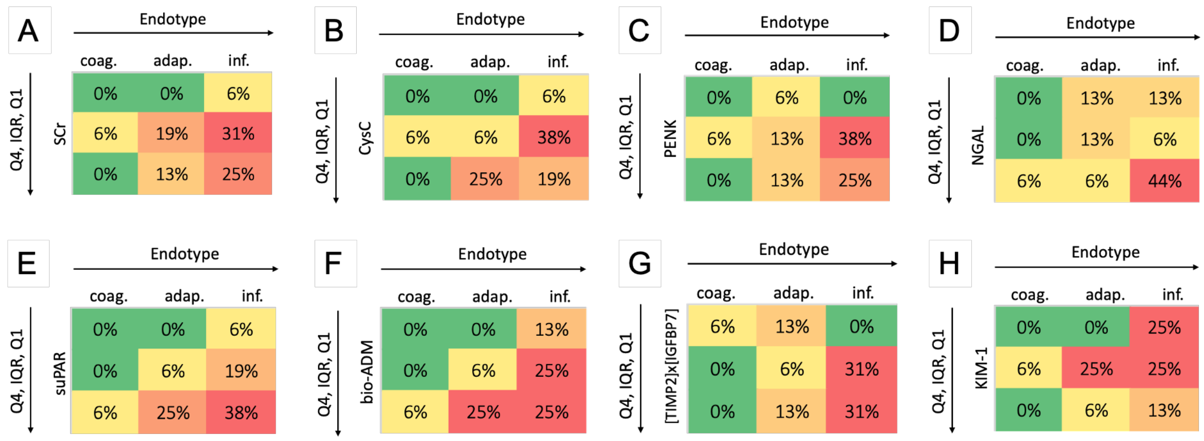

Figure S1: Patients who died within seven days stratified by endotypes and biomarker quartiles. Data are shown as heatmaps. The percentage of patients in the total cohort who died (n=16) is shown in each cell. Left to right: coagulopathic, adaptive, inflammopathic endotype; top to bottom: first quartile, interquartile range, fourth quartile of biomarker values in the entire cohort. Upper panel: A: SCr, serum creatinine; B: CysC, cystatin C; C: PENK, proenkephalin A; D: NGAL, neutrophil gelatinase-associated lipocalin. Lower panel: E: suPAR, soluble urokinase plasminogen activator receptor; F: bio-ADM, bioactive adrenomedullin; G: [TIMP2]x[IGFBP7], the product of urinary tissue inhibitor of metalloproteinases-2 and insulin-like growth factor-binding protein; H: KIM-1, kidney injury molecule-1. IQR, interquartile range.

Stratification for KRT within seven days by biomarker quartiles and endotypes

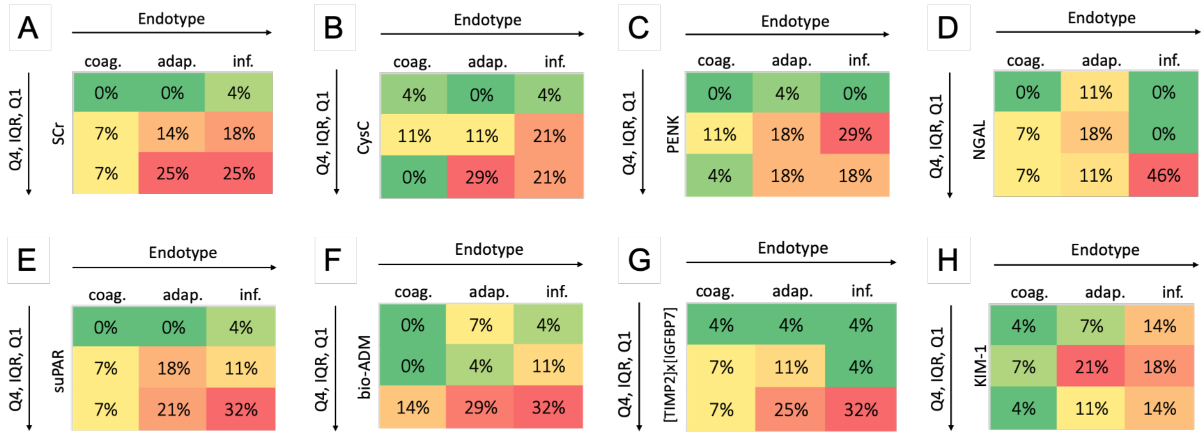

Figure S2: Patients who required kidney replacement therapy (KRT) within seven days stratified by endotypes and biomarker quartiles. Data are shown as heatmaps. The percentage of patients in the total cohort who received KRT (n=28) is shown in each cell. Left to right: coagulopathic, adaptive, inflammopathic endotype; top to bottom: first quartile, interquartile range, fourth quartile of biomarker values in the entire cohort. Upper panel: A: SCr, serum creatinine; B: CysC, cystatin C; C: PENK, proenkephalin A; D: NGAL, neutrophil gelatinase-associated lipocalin. Lower panel: E: suPAR, soluble urokinase plasminogen activator receptor; F: bio-ADM, bioactive adrenomedullin; G: [TIMP2]x[IGFBP7], the product of urinary tissue inhibitor of metalloproteinases-2 and insulin-like growth factor-binding protein; H: KIM-1, kidney injury molecule-1. IQR, interquartile range.

References

1. Sweeney TE, Azad TD, Donato M, Haynes WA, Perumal TM, Henao R, et al. Unsupervised Analysis of Transcriptomics in Bacterial Sepsis Across Multiple Datasets Reveals Three Robust Clusters. Crit Care Med. 2018;46(6):915-25.
